# Clinical significance of potential drug–drug interactions in older adults with psychiatric disorders: a retrospective study

**DOI:** 10.1186/s12888-022-04207-4

**Published:** 2022-08-22

**Authors:** Yu Liu, Man Yang, Yaping Ding, Huanqiang Wang, Hailin Zhang, Dandan Wang, Tianchi Zhuang, Minghui Ji, Yan Cui, Hong Wang

**Affiliations:** 1 Department of Fundamental and Community Nursing, School of Nursing, Nanjing Medical University, Nanjing, Jiangsu Province P.R. China 211166; 2 Department of Nursing Management, Kangda College, Nanjing Medical University, Lianyungang, Jiangsu Province P.R. China 222000; 3 Department of Psychiatry, The Fourth People’s Hospital of Lianyungang, Affiliated to Kangda College, Nanjing Medical University, Lianyungang, Jiangsu Province P.R. China 222003; 4 Department of Occupational Medicine and Environmental Health, Kangda College, Nanjing Medical University, Lianyungang, Jiangsu Province P.R. China 222000; 5 Department of Nursing, The First People’s Hospital of Lianyungang, Affiliated to Kangda College, Nanjing Medical University, Lianyungang, Jiangsu Province P.R. China 222061; 6 Department of Fundamental Nursing, Kangda College, Nanjing Medical University, Lianyungang, Jiangsu Province, P.R. China 222000; 7 Department of Respiratory and Critical Care Medicine, The First Affiliated Hospital with Nanjing Medical University, Nanjing, Jiangsu Province P.R. China 210029

**Keywords:** Potential drug–drug interaction, Adverse drug reaction, Older adults, Psychiatric disorder

## Abstract

**Background:**

Polypharmacy increases the risk of potential drug–drug interactions (pDDIs). This retrospective analysis was conducted to detect pDDIs and adverse drug reactions (ADRs) among older adults with psychiatric disorder, and identify pDDIs with clinical significance.

**Methods:**

A retrospective analysis was carried out based on the medical records of older adults with psychiatric disorders. Data on demographic characteristics, substance abuse, medical history, and medications were extracted. The Lexi-Interact online database was used to detect pDDIs. The minimal clinically important difference (MCID) was set as the change in the Treatment Emergent Symptom Scale (TESS) score between admission and discharge. The median and interquartile ranges were used for continuous variables, and frequencies were calculated for dichotomous variables. Poisson regression was implemented to determine the factors influencing the number of ADR types. The influencing factors of each ADR and the clinical significance of the severity of the ADR were analysed using binary logistic regression. *P* < 0.05 was considered statistically significant.

**Results:**

A total of 308 older adults were enrolled, 171 (55.52%) of whom had at least 1 pDDI. Thirty-six types of pDDIs that should be avoided were found, and the most frequent pDDI was the coadministration of lorazepam and olanzapine (55.5%). A total of 26 ADRs induced by pDDIs were identified, and the most common ADR was constipation (26.05%). There was a 9.4 and 10.3% increase in the number of ADR types for each extra medical diagnosis and for each extra drug, respectively. There was a 120% increase in the number of ADR types for older adults hospitalized for 18–28 days compared with those hospitalized for 3–17 days. There was an 11.1% decrease in the number of ADR types for each extra readmission. The length of hospitalization was a risk factor for abnormal liver function (*P* < 0.05). The use of a large number of drugs was a risk factor for gastric distress (*P* < 0.05) and dizziness and fainting (*P* < 0.05). None of the four pDDIs, including coadministrations of olanzapine and lorazepam, quetiapine and potassium chloride, quetiapine and escitalopram, and olanzapine and clonazepam, showed clinical significance of ADR severity (*P* > 0.05).

**Conclusions:**

pDDIs are prevalent in older adults, and the rate is increasing. However, many pDDIs may have no clinical significance in terms of ADR severity. Further research on assessing pDDIs, and possible measures to prevent serious ADRs induced by DDIs is needed to reduce the clinical significance of pDDIs.

## Background

The prevalence of mental illness increases with population age for people aged 55 years and above [1]. As the pharmacodynamics and pharmacokinetics of drugs in older adults differ from those in younger and middle-aged adults [2], somatic multimorbidity and polypharmacy are prevalent in older adults with severe mental illness [3]. Therefore, the issue of safe medication use in older adults with mental illness deserves attention.

Due to the frequency of multimorbidity among older adults, polypharmacy is also common in this population. Polypharmacy is used to describe multiple, unnecessary, excessive, or unindicated medication consumption [4]. Although there is no standard threshold for the number of medications an individual can use, the regular use of 5 or more medications is widely accepted as the definition of polypharmacy [5, 6]. The prevalence of polypharmacy is reported to range from 7%– to 45% among people aged 65 and above [7], and it is much higher than those of other age groups [8].

Polypharmacy is a key risk factor for potential drug–drug interactions (pDDIs) [9]. Tricyclic antidepressants and selective serotonin reuptake inhibitors (SSRIs) are antidepressants that are often involved in pDDIs [10]. Lexicomp® Drug Interactions is a widely used and comprehensive pDDI knowledge database [11]. pDDIs are classified into several categories according to their potential clinical significance. As databases are updated, pDDIs are constantly updated [12].

pDDIs may induce serious adverse drug reactions (ADRs), and antidepressants are the second most represented subgroup [13]. An ADR is defined as an appreciably harmful or unpleasant reaction resulting from the use of a medication that affects the follow-up medication plan [14].

The risk of pDDIs with antidepressants is inconsistent [15, 16] due to the lack of clinically significant judgements and is based mostly on pharmacokinetics speculation. Clinical significance refers to a large enough size difference between groups for patients to consider the difference important [17] and is usually measured by a minimal clinically important difference (MCID) [18]. For older adults in psychiatric settings, clinical significance refers to serious ADRs that require treatment after drug administration, and psychiatrists usually set the MCID of clinical significance based on the causality between pDDIs and ADRs and the severity of ADR symptoms.

The aim of this study was to identify pDDIs and ADRs in older adults with psychiatric disorders. A secondary aim was to identify pDDIs with clinical significance.

## Materials and methods

### Study design and setting

This was a retrospective study conducted in the Fourth People’s Hospital in Lianyungang which is affiliated with Kangda College of Nanjing Medical University. The hospital is a national third-level specialized hospital and the only specialized hospital for people with mental illness in Lianyungang city, Jiangsu Province, China. It is the provincial judicial psychiatric expertise hospital. The study protocol was approved by the Clinical Research Ethics Committee of the Fourth People’s Hospital of Lianyungang (2021LSYYXLL-P15), and the need for informed consent was waived. All methods were carried out in compliance with the STROBE guidelines.

### Participants and eligibility

The medical records of older adults who were hospitalized in the geriatric psychiatry department and clinical psychology department from July 2nd, 2019 to August 31st, 2021, were checked. The medical records of older adults aged 60 years and over were included. The exclusion criteria were (i) a hospital stay less than 3 days; (ii) less than 5 medication types; and (iii) missing demographic information. When older adults were repeatedly admitted to the hospital for the same major disease, only the latest medical record that met the requirements was included.

### Data extraction and statistical analysis

Collected data included demographic details (sex, age, education level, occupation, marital status, height, weight), substance abuse (alcohol and smoking), medical history (length of onset of disease, number of readmissions, length of hospitalization, major medical diagnosis, total number of medical diagnoses), and medication information (drug, dosage, number of days). Adverse reactions that emerged or were exacerbated after polypharmacy were considered ADRs, and the ADRs recorded as “considered medication-related ADRs” in the medical records were extracted. Only the ADRs of older adults with pDDIs were included in the analysis. In addition, ADRs were recorded based on the Treatment Emergent Symptom Scale (TESS) [19, 20] and the course record recorded by doctors. The TESS has been used in psychiatric settings to assess the severity of ADRs, the link between symptoms and medications, and the measures taken in the form of scores. Behavioural toxicity, laboratory test abnormalities, nervous system, autonomic nervous system, cardiovascular system, and other ADR aspects are included in this scale. Different dosage forms of the same drug prescribed for the same older adult were reported as 1 drug. Record checking and the data extraction process were conducted by two trained researchers.

pDDIs were checked using the Lexi-Interact Online database (https://doctorabad.com/UpToDate/d/di.htm). The coadministration of drugs that should be avoided was considered a severe pDDI and recorded.

In this study, we used the severity of ADRs as an indicator of the clinical significance of pDDIs. There has been no standardization for the clinical significance of ADR severity [21]. The MCID was set based on psychiatrists’ opinions as follows: (total severity score at discharge - total severity score at admission) = 1 or − 1 point. Meeting or exceeding the MCID was considered clinically significant.

Statistical analysis was conducted using IBM SPSS® Statistics Package version 26.0. Normal distributions of continuous variables were tested using the Kolmogorov–Smirnov (K-S) test. Descriptive data were recorded using the median, interquartile ranges and frequencies. Poisson regression analysis was implemented to analyse the influencing factors of the number of ADRs. The clinical significance of the severity of ADRs was analysed using binary logistic regression. The Mann–Whitney (M-W) U test and chi-square test were used to compare differences between groups with or without each ADR, and statistically significant factors were included in the binary regression to analyse the independent influencing factors of the ADRs. The *P* value, odds ratio (OR) and 95% CI were recorded. *P* < 0.05 was considered statistically significant.

## Results

### Baseline characteristics

**Fig. 1:**
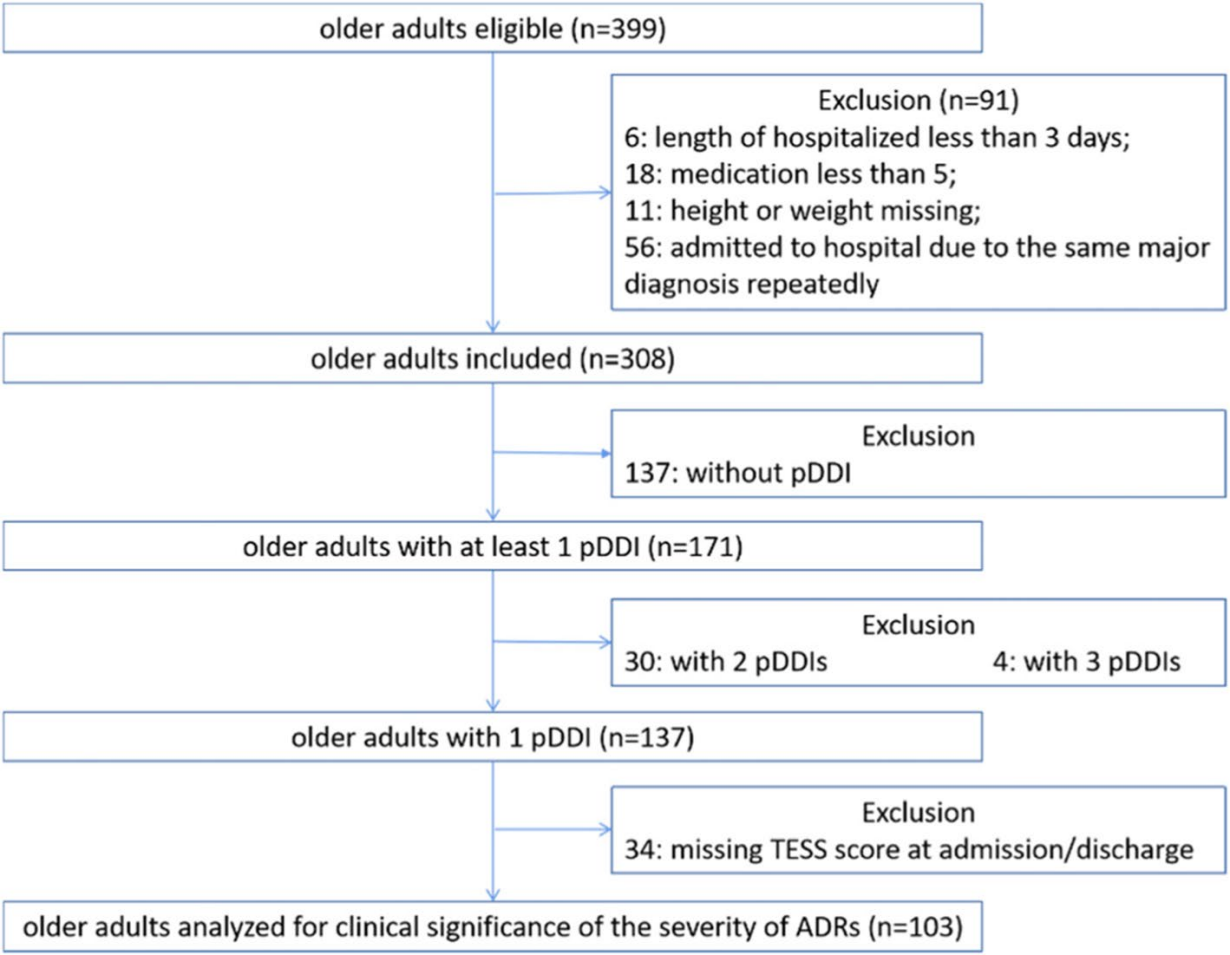
Flow diagram of enrolled older adults

A total of 308 older adults with psychiatric disorders who were aged 60 years and above were enrolled in this study. The screening process is shown in Fig. 1.

Most of the older adults were women (71.1%), married (87.3%), and had a depressive disorder (57.8%). All continuous variables showed a nonnormal distribution. The median age was 68 years old, the median height was 160 cm, and the median weight was 62 kg. The medians of the length of onset of disease, number of drugs, length of hospitalization, readmission and number of medical diagnoses were 36 months, 10, 22 days, once and 3, respectively. The baseline characteristics are summarized in Table 1.

**Table 1:** Demographic and clinical characteristics of older adults

	Total
Number of older adults, n	308
Age (in years), median (IQR)	68 (64–74)
Sex, Female (%)	219 (71.1)
Height (cm), median (IQR)	160 (156–165)
Weight (kg), median (IQR)	62 (55–70)
Length of onset of disease (month), median (IQR)	36 (9–120)
Number of drugs, median (IQR)	10 (8–12)
Length of hospitalization (in days), median (IQR)	22 (15–30)
Number of readmissions, median (IQR)	1 (1–2)
Number of medical diagnoses, median (IQR)	3 (2–5)
Education level, n (%)	
Illiteracy	114 (37.0)
Elementary or junior elementary school	82 (26.6)
Junior high school	60 (19.5)
High school or technical secondary school	32 (10.4)
College or university	20 (6.5)
Marital status, n (%)	
Unmarried	4 (1.3)
Married	269 (87.3)
Divorced	30 (9.7)
Widowed	5 (1.7)
Occupation, n (%)	
Unemployed	192 (62.3)
Retired	87 (28.2)
Farming	22 (7.1)
Others	7 (2.4)
Smokers, n (%)	14 (4.5)
Drinkers, n (%)	14 (4.5)
Major diagnosis, n (%)	
Depressive disorder	178 (57.8)
Organic mental disorder	35 (11.4)
Schizophrenia	25 (8.1)
Bipolar disorder	15 (4.9)
Alzheimer’s disease	14 (4.5)
Dissociative [conversion] disorder	6 (1.9)
Somatoform disorder	6 (1.9)
Others	29 (9.5)

Note: *IQR* Interquartile range

### pDDIs

A total of 3213 prescriptions were found, including 248 types of drugs. Among the 171 (55.52%) older adults who had at least 1 coadministration of drugs that should be avoided, 36 pDDI types were found according to the online database. Drugs for which coadministration should be avoided are listed in Table 2. The coadministration of lorazepam and olanzapine (55.50%), which should be avoided, was the most frequent pDDI in this study.

**Table 2:** Coadministration of drugs that should be avoided

Coadministration of drugs	Number	Frequency (%)
Lorazepam + olanzapine	116	55.50
Olanzapine + potassium chloride	14	6.70
Quetiapine + potassium chloride	14	6.70
Quetiapine + escitalopram	13	6.22
Olanzapine + clonazepam	8	3.83
Olanzapine + piribedil	3	1.44
Quetiapine + piribedil	3	1.44
Quetiapine + paliperidone	3	1.44
Olanzapine + amisulpride	2	0.96
Olanzapine + fluoxetine	2	0.96
Olanzapine + quetiapine	2	0.96
Olanzapine + paliperidone	2	0.96
Risperidone + ziprasidone	2	0.96
Risperidone + amisulpride	2	0.96
Quetiapine + flupentixol and melitroxine	2	0.96
Escitalopram + ziprasidone	1	0.48
Olanzapine + escitalopram	1	0.48
Olanzapine + estazolam	1	0.48
Olanzapine + oxazepam	1	0.48
Olanzapine + flupentixol and melitroxine	1	0.48
Olanzapine + ziprasidone	1	0.48
Diphenhydramine + potassium chloride	1	0.48
Propranolol +rivastigmine	1	0.48
Paliperidone + amisulpride	1	0.48
Clomipramine + potassium chloride	1	0.48
Metoprolol + rivastigmine	1	0.48
Doxepin + potassium chloride	1	0.48
Duloxetine + fluvoxamine	1	0.48
Risperidone + potassium chloride	1	0.48
Quetiapine + amisulpride	1	0.48
Quetiapine + amiodarone	1	0.48
Quetiapine + fluoxetine	1	0.48
Quetiapine + clozapine	1	0.48
Quetiapine + citalopram	1	0.48
Quetiapine + ipratropium bromide	1	0.48
Lorazepam + potassium chloride	1	0.48

### ADRs and associated risk factors

A total of 26 ADRs induced by pDDIs were identified. The most frequent complication was constipation. All ADR types are listed in Table 3.

**Table 3:** Adverse drug reactions induced by pDDIs

Category	Frequency	%
Constipation	31	26.05
Abnormal liver function	14	11.76
Abnormal routine blood examination	8	6.72
Abnormal Electrocardiogram	7	5.88
Hypersomnia	6	5.04
Sweat	6	5.04
Dizziness and fainting	5	4.20
Tachycardia	3	2.52
Dry mouth	2	1.68
Headache	2	1.68
Hypertension	2	1.68
Loss of appetite or anorexia	2	1.68
Akathisia	1	0.84
Decreased activity	1	0.84
Increased salivation	1	0.84
Insomnia	1	0.84
Muscle rigidity	1	0.84
Nausea and vomiting	1	0.84
Tremor	1	0.84
Others		
Fatigue	8	6.72
Flustered	4	3.36
Lower extremity edema	3	2.52
Unsteady walking	3	2.52
Gastric distress	2	1.68
Physical discomfort	2	1.68
Tongue or hand numbness	2	1.68

A total of 171 older adults with pDDIs were included. The analysis of factors affecting the number of ADR types is shown in Table 4. For every extra readmission, the number of ADR types increased by 0.889 times (*P* = 0.010, 95% CI 0.813–0.912). For every extra medical diagnosis, the number of ADR types increased by 1.094 times (*P* = 0.022, 95% CI 1.013–1.181). For every extra drug, the number of ADR types increased by 1.103 times (*P* = 0.001, 95% CI 1.044–1.166). The risk of an increased number of ADR types for older adults who were hospitalized for 18–28 days was 2.200 times (*P* = 0.007, 95% CI 1.241–3.900) that of those who were hospitalized for 3–17 days. A total of 137 older adults with 1 pDDI were analysed for influencing factors of each ADR. Based on the M-W U test, the length of hospitalization was a risk factor for abnormal liver function (*P* = 0.016). The use of a large number of drugs was a risk factor for gastric distress (*P* = 0.026) and dizziness and fainting (*P* = 0.024).

**Table 4:** Influencing factors of the number of ADR types

Item	RC	Group	P	OR	95% CI of OR
Sex	Male	Female	0.305	0.814	0.549–1.207
Age (years)	–	–	0.118	0.979	0.953–1.005
Number of readmissions	–	–	**0.010**	**0.889**	0.813–0.972
Number of medical diagnoses	–	–	**0.022**	**1.094**	1.013–1.181
Number of drugs	–	–	**0.001**	**1.103**	1.044–1.166
Length of hospitalization	3–17 days	18–28 days	**0.007**	**2.200**	1.241–3.900
		29 days and above	0.078	1.736	0.940–3.207

Note: *RC* Reference category

The analysis of influencing factors of constipation and abnormal routine blood examinations is shown in Table 5. These factors were not independent influencing factors of these ADRs (*P* > 0.05). No influencing factors of other ADRs were found (*P* > 0.05).

**Table 5:** Influencing factors of constipation and abnormal routine blood examination

ADR	Factor	P	OR	95% CI of OR
Constipation	Number of medical diagnoses	0.211	1.184	0.909–1.542
	Length of hospitalization	0.178	1.025	0.989–1.063
	Number of drugs	0.154	1.129	0.955–1.335
	Coadministration of lorazepam and olanzapine	0.666	0.752	0.207–2.739
	Coadministration of quetiapine and potassium chloride	0.229	2.694	0.536–13.547
Abnormal routine blood examination	Number of medical diagnoses	0.151	1.326	0.902–1.949
	Number of drugs	0.532	1.087	0.837–1.413
	Coadministration of olanzapine and clonazepam	0.100	5.665	0.717–44.788

### Clinical significance of the severity of ADRs and associated risk factors

Older adults with 1 pDDI were included in this analysis. After excluding those who were missing TESS scores at admission or discharge, 103 patients were analysed in this section. Older adults with TESS scores meeting or exceeding the MCID were assigned to the clinically significant group. Otherwise, they were assigned to the nonclinically significant group.

Sex and age were included to avoid compounding bias. The binary logistic regression of clinical significance is shown in Table 6. The length of hospitalization (*P* = 0.046, OR = 1.184) and the number of ADR types (*P* = 0.002, 10.175) were independent risk factors for the clinical significance of the severity of ADRs. The number of drugs was a protective factor for the clinical significance of the severity of ADRs (*P* = 0.008, OR = 0.493). There was no statistical significance (*P* > 0.05) in the comparison of clinical significance among the four combination groups.

**Table 6:** Influencing factors of the clinical significance of the severity of ADRs

Item	Group	P	OR	95% CI for OR
Sex		0.493	1.911	0.300–12.155
Age		0.280	1.078	0.941–1.234
Number of readmissions		0.219	0.597	0.263–1.359
Number of medical diagnoses		0.419	1.299	0.688–2.454
Length of hospitalization		**0.046**	**1.084**	1.001–1.173
Number of drugs		**0.008**	**0.493**	0.293–0.830
Number of ADR types		**0.002**	**10.175**	2.399–43.157
Coadministration of lorazepam and olanzapine	Yes/No	0.965	0.955	0.121–7.512
Coadministration of potassium chloride and quetiapine	Yes/No	0.998	0.000	0.000-
Coadministration of escitalopram and quetiapine	Yes/No	0.999	0.000	0.000-
Coadministration of clonazepam and olanzapine	Yes/No	0.825	0.702	0.030–16.169

## Discussion

Based on the 3213 prescriptions from the 308 enrolled older adults, 55.52% had at least 1 pDDI. The most frequent pDDI that should be avoided was the coadministration of lorazepam and olanzapine. Constipation was the most common ADR induced by pDDIs. The number of medical diagnoses, the number of drugs used and a length of hospitalization of 18–28 days were risk factors for the number of ADR types; however, readmission was a protective factor for the number of ADR types. The length of hospitalization was a risk factor for abnormal liver function, and the number of drugs was a risk factor for gastric distress and dizziness and fainting. The length of hospitalization and the number of ADR types were risk factors for the clinical significance of the severity of ADRs. The number of drugs was a protective factor for the clinical significance of the severity of ADRs. Coadministrations of lorazepam and olanzapine, quetiapine and potassium chloride, quetiapine and escitalopram, and olanzapine and clonazepam were not risk factors for the clinical significance of the severity of ADRs.

pDDIs are prevalent in older adults with psychiatric disorders, and the rate is increasing. This finding is similar to those of other studies. Ocana-Zurita [22] performed a retrospective and cross-sectional study and found that 68.25% of schizophrenic patients were at risk of pDDIs. Ruangritchankul [23] found that 76% of older adults diagnosed with dementia experienced at least 1 pDDI.

Independent factors of ADRs vary in studies. de Vries [24] performed a cohort study and found that polypharmacy was a risk factor for ADRs. O′Mahony [25] identified that 8 factors, including female sex and having 4 or more multimorbidities were independent risk factors for ADRs. However, Lee [26] performed a meta-analysis and found that sex did not significantly influence the incidence of ADRs. Sun [27] conducted a study and found that 96.8% of ADRs occurred within 14 days of hospitalization, and length of stay, the number of drugs used in the hospital and underlying basic diseases were independent risk factors for ADRs. However, Lavan [28] did not identify associations between ADRs and age, sex, the number of daily medication or length of stay. The difference in results may be due to different populations and different types of drugs.

Many pDDIs may not be clinically significant in terms of the severity of ADRs caused by pDDIs. Madhusoodanan [29] conducted a pilot study and found that coadministration of lorazepam and olanzapine caused no adverse consequences. Bergemann [30] discovered that after the coadministration of olanzapine and lorazepam, the dose-corrected olanzapine plasma concentration was no different from the plasma levels under olanzapine monotherapy. However, a case report showed that IM olanzapine and IM lorazepam lowered blood pressure and caused dizziness [31].

The probable reasons are as follows. First, pDDIs are mainly speculated based on drug pharmacokinetic features, and psychotropic drugs are usually metabolized by several enzymes that reduce the risk of pDDIs [16]. Second, the route of administration may affect pDDIs and ADRs. The occurrence of pDDIs may be lower for the oral administration of olanzapine and lorazepam according to the database. In this study, antipsychotics, antidepressants and benzodiazepines were all orally administered and the incidence of ADRs was lowered. Third, the data are biased. For example, there were no ADRs among any older adults who used quetiapine in combination with escitalopram. Fourth, the majority of ADRs were mild [32] and preventable [33]. Psychiatrists have taken pDDIs seriously, and have prevented ADRs by controlling drug doses, strengthening monitoring, and taking measures in advance.

### Strengths and limitations

The strength of this study is that we discussed pDDIs and ADRs not only from a statistical perspective but also from a clinical significance perspective. Considering clinical medication safety, we analysed the relationship between the coadministration of drugs and ADRs.

Our work has five limitations. First, there is no standardization for clinical significance, and none of the approaches to set the MCID are ideal. This was a retrospective study, and other more objective calculation methods for establishing the MCID, such as anchor-based methods and triangulation of methods, could not be applied. Another limitation would be since this was a retrospective study and the causality of ADRs was not routinely assessed, the causal relationship between ADRs and the coadministration of drugs may not be accurate. Moreover, we focused on older adults with a major diagnosis of a psychiatric disorder who were taking central nervous system drugs. The fourth limitation is that we extracted information from the long-term physicians’ orders and ignored temporary physicians’ orders. Lastly, only four combinations of drugs were analysed in this study, and other combinations were ignored due to the low number of cases.

## Conclusion

Above all, pDDIs were prevalent in older adults with psychiatric disorders. From the perspective of coadministration inducing severe ADRs, four combinations were not clinically significant in this study. Further research on assessing pDDIs and possible measures to prevent ADRs induced by DDIs is needed to reduce the clinical significance of pDDIs.

## Data Availability

The datasets used and/or analyzed during the current study are available from the corresponding author on reasonable request.

## Abbreviations

pDDI: Potential drug-drug interaction
SSRI: Selective serotonin reuptake inhibitors
ADR: Adverse drug reaction
MCID: Minimally clinical important difference
TESS: Treatment Emergent Symptom Scale
